# Actin-dependent membrane polarization reveals the mechanical nature of the neuroblast polarity cycle

**DOI:** 10.1016/j.celrep.2021.109146

**Published:** 2021-05-18

**Authors:** Bryce LaFoya, Kenneth E. Prehoda

**Affiliations:** 1 Institute of Molecular Biology, Department of Chemistry and Biochemistry, University of Oregon, Eugene, OR 97403, USA; 2 Lead contact

## Abstract

The Par complex directs fate-determinant segregation from the apical membrane of asymmetrically dividing *Drosophila* neuroblasts. While the physical interactions that recruit the Par complex have been extensively studied, little is known about how the membrane itself behaves during polarization. We examined the membrane dynamics of neuroblasts and surrounding cells using a combination of super-resolution and time-lapse imaging, revealing cellular-scale movements of diverse membrane features during asymmetric division cycles. Membrane domains that are distributed across the neuroblast membrane in interphase become polarized in early mitosis, where they mediate formation of cortical patches of the Par protein atypical protein kinase C (aPKC). Membrane and protein polarity cycles are precisely synchronized and are generated by extensive actin-dependent forces that deform the surrounding tissue. In addition to suggesting a role for the membrane in asymmetric division, our results reveal the mechanical nature of the neuroblast polarity cycle.

## In brief

LaFoya and Prehoda examine the membrane dynamics of asymmetrically dividing *Drosophila* neuroblasts and discover that the membrane undergoes a polarity cycle. Their studies show that membrane and protein polarity is precisely correlated and that cellular-scale forces generated during the cycle significantly deform the surrounding tissue.

## Graphical abstract

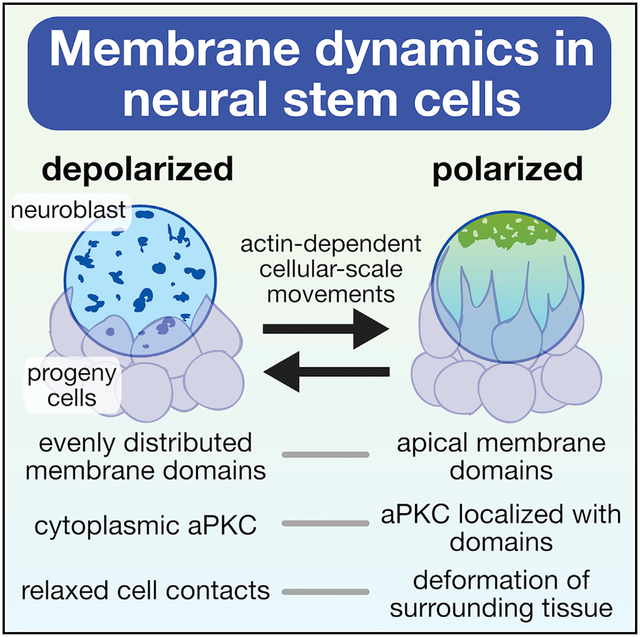

## INTRODUCTION

The Par polarity complex mediates functions such as directional transport and fate-determinant segregation in diverse animal cells (Lang and Munro, 2017; Riga et al., 2020; Venkei and Yamashita, 2018). A key step in Par complex function is formation of the Par domain, a continuous region along the cell membrane containing the Par complex proteins Par-6 and atypical protein kinase C (aPKC). Formation of the Par domain is a highly dynamic process involving recruitment from the cytoplasm and movement along the membrane (Gubieda et al., 2020; Illukkumbura et al., 2020; Sunchu and Cabernard, 2020). In asymmetrically dividing *Drosophila* neuroblasts, for example, initially cytoplasmic Par-6 and aPKC accumulate in the membrane’s apical hemisphere early in mitosis (Wodarz et al., 2000; Petronczki and Knoblich, 2001; Rolls et al., 2003; Homem and Knoblich, 2012). Membrane recruitment is followed by coalescence of the proteins into a cortical region concentrated around the apical pole (Oon and Prehoda, 2019). Extensive effort has been directed toward deciphering the physical interactions that recruit Par-6 and aPKC from the cytoplasm to the membrane. Here, we examine the dynamics of the neuroblast membrane with the goal of understanding its potential role in the polarity cycle and also to determine whether membrane dynamics might provide insight into the mechanism of formation of the Par domain.

While current models for neuroblast polarity focus on the protein-protein interactions that recruit the Par complex to the membrane (Knoblich, 2010; Lang and Munro, 2017; Venkei and Yamashita, 2018), accumulating evidence indicates that additional mechanisms may contribute to polarity. Neuroblast polarization is a stepwise process in which membrane targeting of the Par protein aPKC to the apical hemisphere is followed by coordinated movements toward the apical pole (Oon and Prehoda, 2019). Coalescence of aPKC requires the actin cytoskeleton (Oon and Prehoda, 2019), as does maintenance of the polarized state (Hannaford et al., 2018; Oon and Prehoda, 2019). However, the role of the actin cytoskeleton in the process remains unclear, as myosin II has been reported to be dispensable for apical polarity (Barros et al., 2003) and instead directly participates in polarization of the basal cortex (Barros et al., 2003; Hannaford et al., 2018). The potential lack of a role for myosin II in apical neuroblast polarity suggests that the actin cytoskeleton could play a passive role in contrast to the *C. elegans* zygote, for example, where actomyosin-generated cortical flows are essential for segregation of the Par complex along the membrane (Munro et al., 2004; Wang et al., 2017). Thus, while the actin cytoskeleton is required for neuroblast polarity, the extent to which force is generated during the polarity cycle has been unclear.

The plasma membrane plays a central role in Par-mediated polarity by establishing the scaffold for formation of the Par domain, yet its precise role in polarity has not been elucidated. In polarized cells such as epithelia, localized accumulations of specific phospholipids are thought to be important for formation of the Par domain, although the existence and function of these microdomains is controversial (van IJzendoorn et al., 2020; Riga et al., 2020; Stone et al., 2017). In the *C. elegans* zygote, a clear membrane asymmetry emerges during formation of the Par domain with filopodia-like structures preferentially forming in the anterior domain (Scholze et al., 2018; Hirani et al., 2019). What little is known about the neuroblast membrane suggests that it is relatively featureless, as several phosphoinositide sensors have been reported to localize uniformly to the membrane (Doyle et al., 2017; Koe et al., 2018; Loyer and Januschke, 2018). Here, we investigate whether the neuroblast membrane contains any heterogeneities and, if so, whether these features might provide insight into the polarity cycle.

## RESULTS

### The neuroblast membrane is heterogeneous and forms extensive contacts with progeny cells

**Figure 1.**
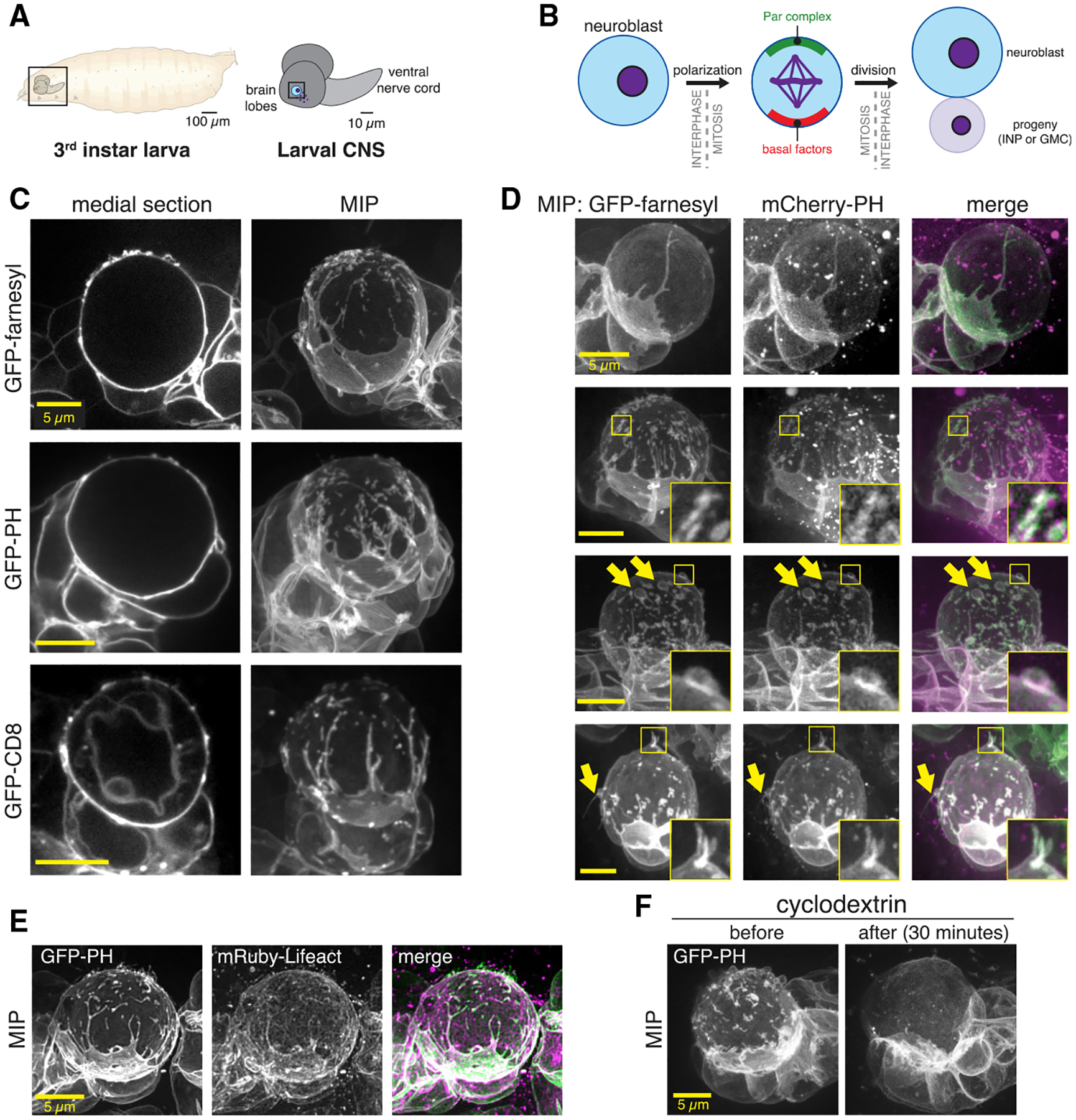
The neuroblast membrane is heterogenous (A) *Drosophila* larval central nervous system explants used in this study. (B) Larval brain neuroblast polarity cycle. Division results in self-renewal of the apical daughter and production of an intermediate neuronal precursor (INP) or ganglion mother cell (GMC). (C) Super-resolution images of neuroblasts and their progeny from *Drosophila* third instar larvae. Neuroblasts expressed the indicated membrane marker under the control of the UAS promoter and driven by worniu-GAL4. A single medial optical section is shown along with a maximum intensity projection (MIP) made from optical sections through the neuroblast’s front hemisphere. Rotations of each cell’s full volume MIP are shown in Video S1. (D) Direct comparison of neuroblast features using simultaneous imaging of multiple membrane markers. Images are MIPs as in (C). Selected features are highlighted with arrows and boxes with magnified inset. Rotations of each cell’s full volume MIP are shown in Video S2. (E) Colocalization of neuroblast membrane features (GFP-PH) and actin (mRuby-Lifeact). (F) Effect of cyclodextrin treatment on neuroblast membrane heterogeneity. MIPs constructed from optical sections taken from the same neuroblast before and 30 min following treatment with cyclodextrin are shown.

We examined the neuroblast membrane using three markers, each of which interacts with the membrane via a distinct mechanism: a farnesyl-modified peptide that integrates into the bilayer (FP-farnesyl) (Zhou et al., 2015); the Pleckstrin Homology domain from PLCδ that interacts with the headgroup of the phosphoinositide PI(4,5)P_2_ (FP-PH) (Verstreken et al., 2009); and the integral membrane protein CD8 that traverses the bilayer (FP-CD8) (Lee and Luo, 1999). We expressed fluorescent protein fusions of the markers specifically in the neuroblast lineage using the upstream activating sequence (UAS) promoter and worniu-GAL4 driver and imaged actively dividing central nervous system neuroblasts from third instar larval brain explants (Figures 1A and 1B). To image neuroblast membranes at super-resolution, we used spinning-disk confocal microscopy with optical photon reassignment (Azuma and Kei, 2015). Each of the membrane markers outlined the plasma membrane of neuroblasts and their smaller progeny cells, with little background from other cells (Figure 1C; Video S1). For FP-farnesyl and FP-PH, the plasma membrane was marked nearly exclusively (small vesicle-like signals were occasionally seen with each) whereas FP-CD8 also marked some internal membranes.

Individual optical sections and maximum intensity projections (MIPs) of the full cell volume revealed a rich landscape of neuroblast membrane features, including blebs and filopodial-like extensions, smaller domains, and extensive contacts with progeny cells (Figures 1C and 1D; Videos S1 and S2). Extensions were typically greater than 0.5 μm in length whereas membrane domains were smaller areas of marker enrichment that were closer to the cell body but also protruded to some extent. The membrane markers also revealed that progeny cells form extensive contacts with their associated neuroblast. These contacts were large in surface area and also typically included filopodial extensions that emanated from the progeny cell toward the opposite side of the neuroblast. In many instances, the progeny filopodia encircled the neuroblast to an extent that the progeny appeared to engulf part of the neuroblast. We observed the same membrane features when simultaneously imaging distinct membrane markers (Figure 1D), indicating that the markers report on the same set of features. Furthermore, membrane features were associated with cortical F-actin, as detected by the Lifeact sensor (Figure 1E).

The smaller membrane domains dissipated when methyl-β-cyclodextrin was included in the surrounding media (Figure 1F; n = 25 neuroblasts). Cyclodextrin sensitivity and CD8 enrichment are characteristics of membrane microdomains – localized areas where specific phospholipids and proteins are concentrated (Barman and Nayak, 2007; Pang et al., 2007), although the increased membrane marker signal may be due to increased amounts of plasma membrane, consistent with their slight protrusion above the cell surface (Figures 1A–1E) and similar observations in the worm zygote (Hirani et al., 2019).

### The neuroblast membrane undergoes an actin-cytoskeleton-dependent polarity cycle

**Figure 2.**
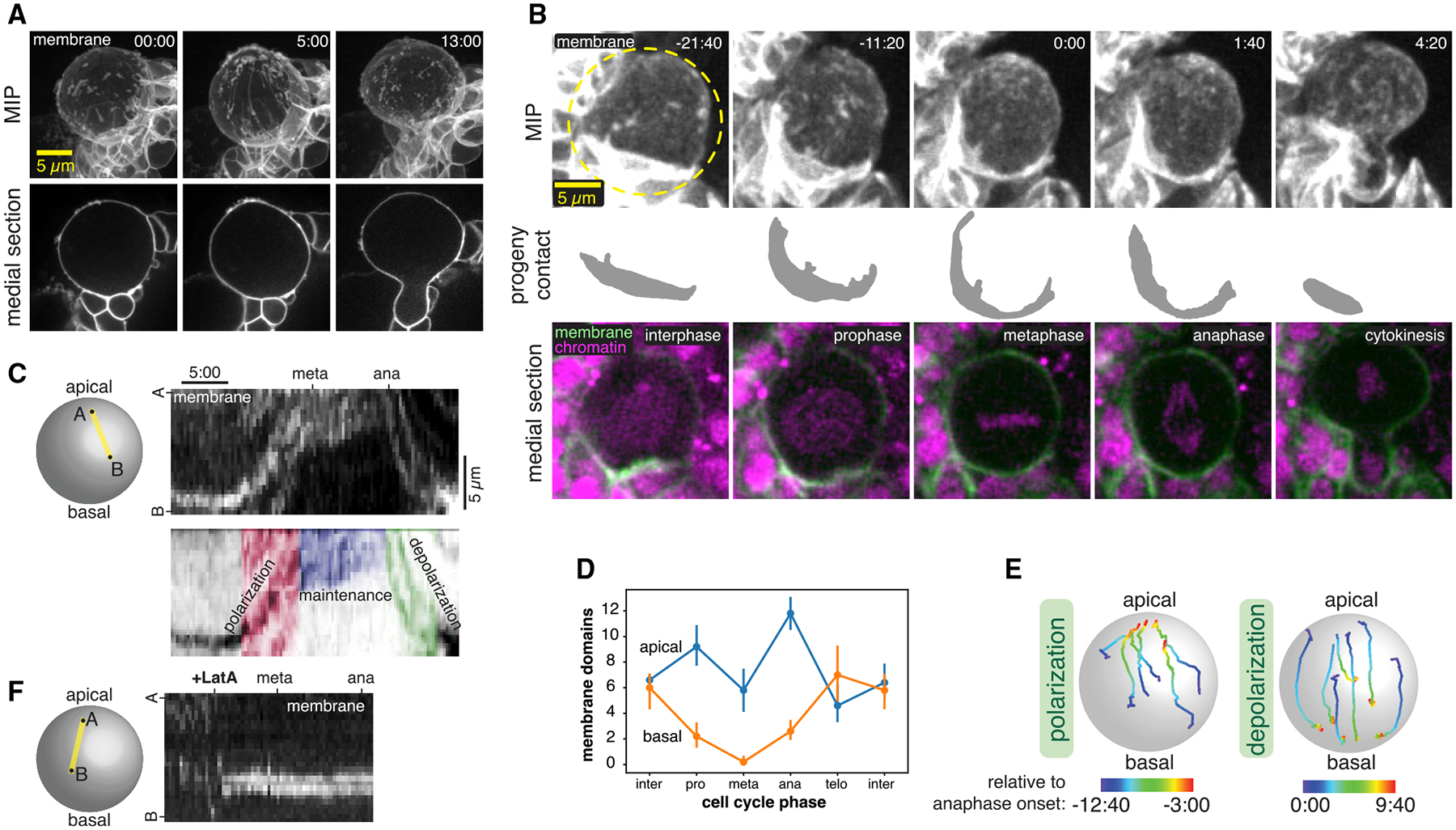
The neuroblast membrane undergoes a polarity cycle (A) Membrane dynamics of a dividing neuroblast and progeny cells observed with super-resolution imaging of GFP-farnesyl (“membrane”). A single medial optical section is shown along with a MIP of optical sections through the front hemisphere. Time in minutes and seconds relative to when the first frame was collected is shown in each frame. (B) Neuroblast membrane dynamics through a full division cycle. Selected frames from Video S3 are shown in MIP of the front hemisphere GFP-PH signal (“membrane”), with a manual segmentation of the basal daughter cell membrane that contacts the neuroblast (progeny contact), and a medial section with both GFP-PH and RFP-H2A (“chromatin”) signals. Time in minutes and seconds relative to anaphase onset is indicated in the upper-right corner of each frame. (C) Kymograph along the indicated axis following the progression of a GFP-PH membrane domain over the course of mitosis (from Video S3). A legend showing the three phases of dynamics is shown below the kymograph. (D) Membrane domain count in the apical and basal hemispheres at different cell cycle phases (n = 5 different neuroblasts; bars represent one standard deviation). Note that the total domain count decreases at metaphase because the apical domains coalesce. (E) Particle tracking of individual membrane domains during both the polarization and depolarization phases from Video S3. The color of each track is coded by the time relative to anaphase onset. (F) Kymograph along the indicated axis following the progression of a GFP-PH membrane domain over the course of mitosis in a LatA-treated neuroblast (from Video S4A).

To examine neuroblast membrane dynamics, we acquired time series of optical sections across the cell volume approximately once per 5 min, a frequency that did not cause significant photobleaching using the optical reassignment imaging method. Even at this temporal resolution, MIPs of sections through the cell’s full volume revealed changes in the membrane over the course of the cell cycle (Figure 2A). Membrane dynamics were not constant, with relatively little change until sometime in mitosis, when large movements appeared to begin before cleavage furrow ingression and end shortly after the completion of division.

To follow the neuroblast membrane at higher temporal resolution, we used standard-resolution spinning disk confocal microscopy, imaging the full cell volume every 20 s. Additionally, we simultaneously imaged the chromosomal marker RFP-His2A to more precisely identify the cell-cycle stages at which transitions in membrane dynamics occur. From the resulting videos, we identified four distinct temporal phases of neuroblast membrane movements (Figures 2B and 2C; Video S3). In interphase, membrane features exhibited only small movements that were uncoordinated and lacked any clear directionality. The relative calm of the interphase membrane gave way to a period of highly coordinated dynamics in early prophase in which features across the cell membrane moved rapidly toward the apical pole. We detected apical movement not only in features such as localized marker enrichments, but also in progeny cell contacts that are typically near the basal pole, indicating that sub-stantial force accompanies these movements (Figure 2B; Video S3). Continued apical movement during prophase ultimately led to accumulation of membrane features in the apical hemisphere of the cell (i.e., polarization) until this phase ends near metaphase (Figures 2B–2E).

The polarized membrane state was maintained for a short period until anaphase began and membrane movements reversed direction, leading to their dispersal across the membrane surface (i.e., depolarization) by the completion of cytokinesis. The basally directed movements that occur in anaphase also caused the progeny cell contacts at the basal region of the neuroblast membrane to relax to their pre-polarization state (Figure 2B; Video S3). Thus, there are four phases of membrane dynamics during neuroblast asymmetric cell division: (1) a relatively static interphase with features evenly distributed across the cell surface and (2) apically directed movement during prophase that generates (3) an apically polarized state that is maintained through metaphase and (4) a depolarization phase during anaphase. The process that drives membrane dynamics operates at the cellular scale, altering both the basal and apical membranes, and the force generated during the process significantly deforms the surrounding tissue.

To gain insight into the underlying process that drives membrane movements, we examined membrane dynamics in neuroblasts treated with the actin depolymerizing drug Latrunculin A (LatA). As shown in Figure 2F and Video S4, membrane domains halt apically directed movements during prophase immediately following LatA exposure and become nearly completely static (n = 15 neuroblasts). However, while the dynamics of membrane domains were dependent on the actin cytoskeleton, the domains themselves were unaffected by LatA treatment. Simultaneous imaging of GFP-PH and mRuby-Lifeact confirmed that membrane movements ceased when cortical actin dissipated (Video S4). Thus, an intact actin cytoskeleton is required for the complex dynamics of membrane domains during asymmetric cell division, but the domains persist in the absence of F-actin.

### The Par protein aPKC localizes to membrane domains and follows their dynamics

**Figure 3.**
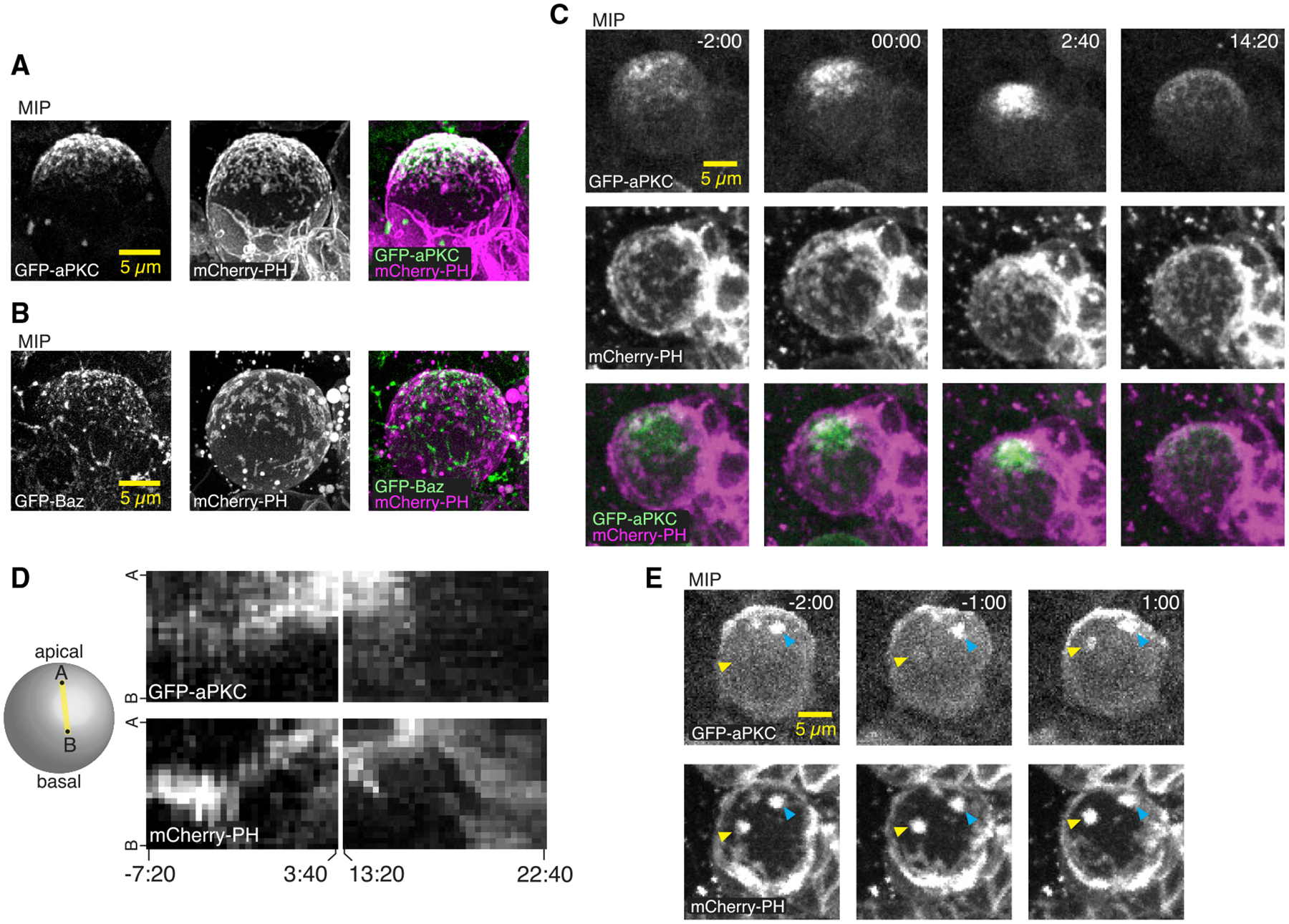
Neuroblast membrane and polarity protein dynamics are coupled (A) MIP constructed from super-resolution optical sections of the polarity proteins aPKC and the mCherry-PH membrane marker. (B) MIP of Bazooka (Baz; aka Par-3) and mCherry-PH as in (A). (C) Simultaneous imaging of membrane and aPKC dynamics during neuroblast asymmetric division. Selected frames from Video S5 of a neuroblast expressing GFP-aPKC from its endogenous promoter and mCherry-PH via worniu-GAL4. Time relative to nuclear envelope breakdown (NEB) is shown. As described previously, aPKC accumulates on the apical membrane beginning in early prophase, followed by a coalescence phase shortly before NEB. Membrane features are present throughout the cell cycle, and their movements occur simultaneously with aPKC. (D) Kymograph along the apical-basal axis of Video S5 showing the correlated dynamics of aPKC and the neuroblast membrane during polarization and depolarization. (E) Selected frames from Video S6 showing an example of aPKC recruitment to a membrane patch as it moves from the basal to the apical hemisphere. Yellow arrowheads mark the membrane patch and the corresponding aPKC signal. Cyan arrowheads mark a membrane patch and corresponding aPKC signal that starts and ends in the apical hemisphere.

The phases of neuroblast membrane dynamics we observed, rapid polarization and then depolarization, resemble those of Par polarity proteins such as Bazooka (Baz; a.k.a. Par-3) and aPKC (Oon and Prehoda, 2019), with the key difference being that membrane domains are present throughout the cell cycle, whereas aPKC is cytoplasmic in interphase and does not begin accumulating on the membrane until early prophase (Hannaford et al., 2018; Oon and Prehoda, 2019). We first examined the localization of the aPKC and Baz using super-resolution imaging and found that while aPKC significantly overlapped with the membrane domains, Baz was predominantly localized away from domains (Figures 3A and 3B). We therefore examined whether membrane and aPKC dynamics are correlated by imaging larval brain neuroblasts expressing GFP-aPKC from its endogenous promoter and *worniu-GAL4*-driven mCherry-PH. We observed accumulation of aPKC early in mitosis, with localized enrichments (i.e., patches) forming at apically localized membrane domains and aPKC that was more diffusely distributed elsewhere in the apical hemisphere (Figure 3; Video S5). Both pools of cortical aPKC, membrane-domain-localized patches and diffuse, moved toward the apical pole simultaneously, with protein and membrane movement beginning simultaneously (i.e., within one frame; n = 8 neuroblasts). The membrane domains often appeared to encircle the aPKC (Figures 3A and 3C; Video S5), and membrane domains that were initially in the basal hemisphere did not recruit aPKC until they passed the equator into the apical hemisphere (Figure 3E; Video S6; n = 3). Upon completion of the polarization process, membrane domains and aPKC remained tightly localized around the apical pole for a short period before disassembling via basally directed movements. Disassembly of the polarized membrane domains and aPKC began simultaneously (within one frame; n = 8 neuroblasts), as both moved rapidly toward the emerging cleavage furrow. Following disassembly, aPKC signal rapidly dissipated from the membrane, whereas the depolarized membrane domains persisted into the following interphase. Thus, aPKC patches form at apically localized membrane domains, and both populations of cortical aPKC—patches and diffuse—precisely follow membrane dynamics with the same coordinated apically and basally directed movements during prophase and anaphase, respectively.

### Neuroblast membrane domains mediate aPKC cortical patch formation

**Figure 4.**
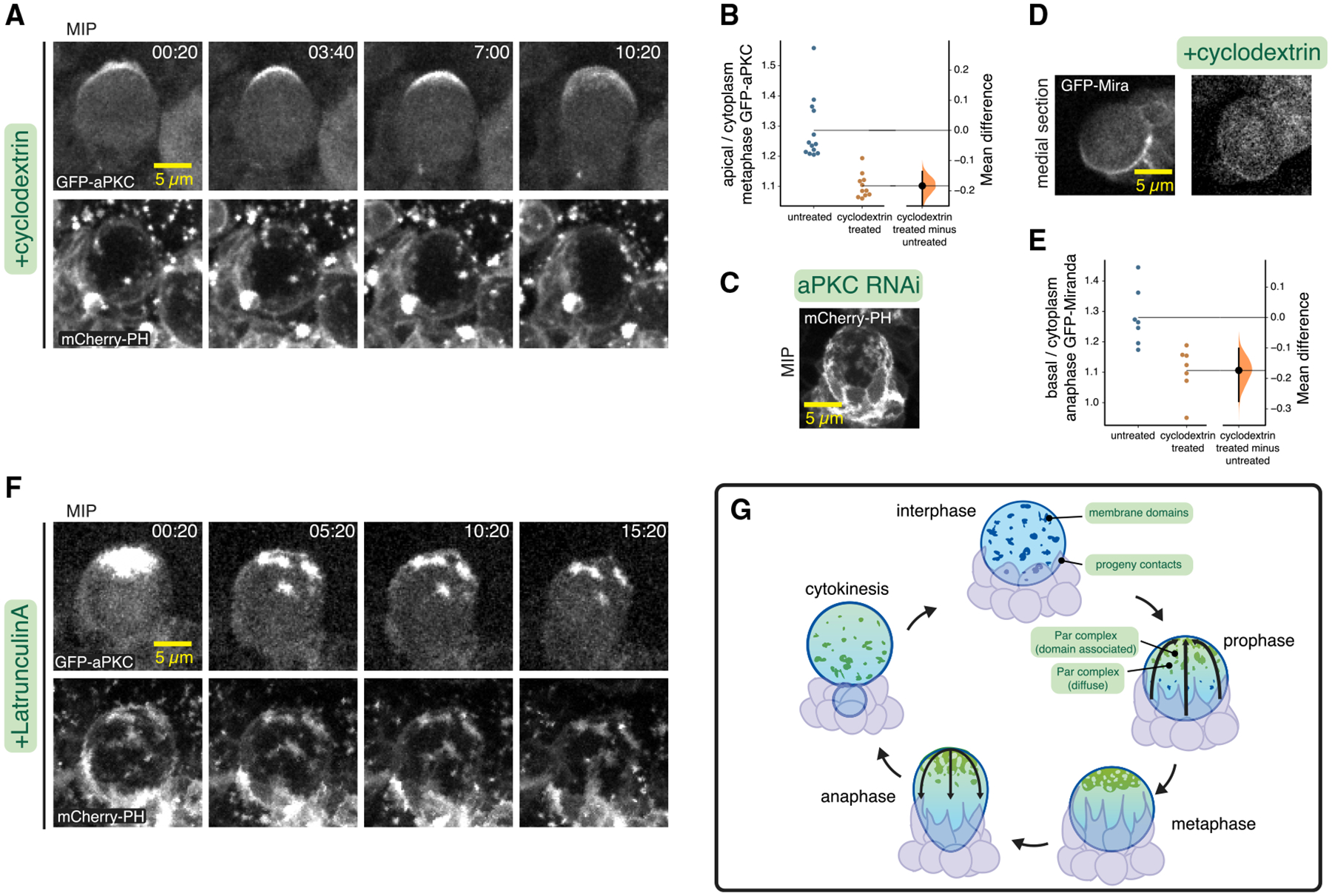
Membrane domains mediate aPKC polarity maintenance (A) Membrane and aPKC dynamics in a neuroblast in which membrane domains were removed via cyclodextrin treatment. Selected frames subsequent to polarization are shown from Video S7. Time is shown in minutes and seconds relative to NEB. (B) Gardner-Altman estimation plot of the effect of cyclodextrin on the cortical recruitment of aPKC. Apical cortical to cytoplasmic signal intensity ratios of GFP-aPKC are shown for individual metaphase neuroblasts treated with cyclodextrin. Statistics: bootstrap 95% confidence interval. (C) Neuroblast expressing mCherry-PH and aPKC RNAi. Selected frame from Video S8. (D) Comparison of GFP-Miranda in untreated and cyclodextrin-treated neuroblasts (anaphase) shown in medial sections. MIPs for the same neuroblasts are shown in Video S9. (E) Gardner-Altman estimation plot of the effect of cyclodextrin on the cortical recruitment of Miranda. Basal cortical to cytoplasmic signal intensity ratios of GFP-Miranda are shown for individual anaphase neuroblasts treated with cyclodextrin. Statistics: bootstrap 95% confidence interval. (F) Membrane and aPKC maintenance dynamics in a neuroblast in which the actin cytoskeleton was disrupted by treatment with LatA. Selected frames following LatA addition are shown from Video S10. (G) Model for neuroblast membrane dynamics. Membrane features are dispersed over the entire cell surface during interphase and progeny cell contacts are typically restricted to the basal pole. Apically directed movements begin in prophase, when aPKC is recruited to the apical hemisphere of the membrane (enriched at membrane domains and diffuse elsewhere), and movements lead to metaphase polarization and deformation of progeny contacts. Basally directed movements begin in anaphase and depolarize membrane features and return progeny cell contacts to the basal hemisphere.

The targeting of aPKC cortical patches to membrane domains and the correlated movement of membrane and polarity protein suggest that the two may be functionally related. We tested the effect of removing membrane domains on the cortical localization of aPKC by examining aPKC localization in neuroblasts that lacked membrane domains owing to cyclodextrin treatment. These neuroblasts failed to form patches of aPKC, with the remaining cortical aPKC signal diffusely distributed over the apical cortex (n = 12; Figure 4A; Video S7). The diffuse aPKC continued to polarize at the apical pole in the absence of membrane domains as it did in untreated neuroblasts (Figure 3; Video S5), although the overall signal was significantly reduced in the cyclodextrin-treated neuroblasts (Figure 4B). We also tested whether aPKC is required for membrane domains to form and noticed no effect on membrane structure in neuroblasts expressing an RNAi directed against aPKC (Figure 4C; Video S8).

To determine if the loss of cortical patches influenced aPKC’s ability to regulate polarity, we examined the localization of the basal factor Miranda in cyclodextrin-treated neuroblasts. Miranda is normally polarized to the basal cortex due to the activity of aPKC, leading to its segregation into the basal daughter cell during cytokinesis (Rolls et al., 2003). Although Miranda polarization was still detectable in cyclodextrin-treated neuroblasts, its basal localization at anaphase was significantly reduced compared to untreated neuroblasts (Figures 4D and 4E; Video S9). Taken together, the effects of cyclodextrin on aPKC and Miranda indicate that membrane domains are required for cortical aPKC patches, and aPKC and Miranda polarity are significantly reduced without patches. Importantly, the diffusely cortical aPKC that remains in the absence of membrane domains is polarized similarly (as are the progeny cell contacts), suggesting that the underlying process that drives coalescence does not require the domains.

The actin cytoskeleton is required to maintain the diffusely localized pool of cortical aPKC in the apical hemisphere (Hannaford et al., 2018; Oon and Prehoda, 2019). We examined whether the membrane-domain-localized cortical aPKC patches also require F-actin to retain aPKC. In LatA-treated neuroblasts expressing GFP-aPKC and mCherry-PH as a membrane marker, we observed rapid dissipation of cortical aPKC that was not co-localized with a membrane domain (signal below limit of detection in 24.9 ± 10 min; n = 21; Figure 4F; Video S10), as previously described (Oon and Prehoda, 2019). However, aPKC patches remained localized to membrane domains for very extended time periods in all neuroblasts examined (38.7 ± 6 min; many neuroblasts had signal remaining at the end of the imaging session; n = 21), indicating that their retention in the apical hemisphere does not require the actin cytoskeleton. Thus, the two pools of cortical aPKC are maintained in the apical hemisphere via an actin-dependent mechanism for diffusely localized aPKC and a membrane-structure-dependent one for cortical aPKC patches.

## DISCUSSION

We examined the neuroblast membrane using super-resolution imaging and found that it is very heterogenous, with features such as small cyclodextrin-sensitive domains, filopodial extensions, and extensive contacts with progeny cells (Figure 1; Videos S1 and S2). Membrane domains are initially dispersed across the cell surface in interphase, with progeny contacts typically near the basal pole. The membrane undergoes several phases of coordinated movements during mitosis (Figure 4G), first polarizing by enriching features in the apical hemisphere with coordinated, apically directed movements that are dependent on the actin cytoskeleton. The forces that generate the polarized neuroblast membrane deform the surrounding tissue, indicating that they are significant in magnitude and scale. After a brief phase where the polarized state is maintained, basally directed movements depolarize the membrane by redistributing the features across the cell surface. We found that these complex dynamics, including the timing of the transitions between phases and the characteristics of the movements, are precisely correlated with those of the Par polarity protein aPKC (Figure 3; Video S5). Furthermore, membrane domains participate in the recruitment and retention of aPKC, specifically recruiting aPKC when they are in the apical hemisphere (Figure 3E; Video S6) and removing domains with cyclodextrin significantly reduces both apical and basal polarity (Figures 4B and 4E).

The behavior of the membrane indicates that the neuroblast polarity cycle is a mechanical process in which cellular-scale forces are generated. Par proteins were recently found to undergo apically directed movements that lead to neuroblast polarization (Oon and Prehoda, 2019), but whether the underlying cellular process that drives these movements acts locally or on a larger scale has not been known. We observed movement of membrane features in the apical hemisphere but also near the basal pole, including the extension of filopodial-like arms from progeny cells that wrap around the neuroblast. The deformation of the surrounding tissue raises the possibility that the polarity cycle could be driven by contacting cells rather than cell autonomously by the neuroblast, as has been observed in some epithelial cells (Pohl et al., 2012; Roh-Johnson et al., 2012). However, we believe two features of the data strongly support a cell-autonomous model: membrane domains distant from progeny contacts move with the same dynamics, and the polarity cycle is tightly coupled to the neuroblast’s cell cycle (Figure 2; Video S3).

The membranes of both the *Drosophila* neuroblast (present work) and the *C. elegans* zygote (Scholze et al., 2018; Hirani et al., 2019) are polarized during asymmetric division. The zygote membrane is initially devoid of features, and while some movement occurs, it is primarily polarized by the preferential appearance of filopodial-like structures in the anterior domain. In contrast, features are present on the neuroblast membrane throughout the cell cycle, and polarization results from the coordinated movement of membrane domains toward the apical pole (Figure 2). In the zygote, pulsatile contractions of actomyosin generate cortical flows important for Par polarity. We have found that an intact actin cytoskeleton is necessary for neuroblast membrane polarity (Figure 2). However, myosin II has been reported to be directly involved in basal, not apical, neuroblast polarity (Barros et al., 2003; Hannaford et al., 2018), and while myosin II has been extensively imaged in the neuroblast (Barros et al., 2003; Cabernard et al., 2010; Connell et al., 2011; Koe et al., 2018; Roth et al., 2015; Roubinet et al., 2017; Tsankova et al., 2017), cortical actomyosin dynamics during polarization have not been reported. Future work will be directed at understanding how the extensive forces that occur during the neuroblast polarity cycle are generated.

Our results indicate that the neuroblast membrane plays a role in polarity initiation and maintenance. We observed recruitment of aPKC to apical membrane domains and retention of aPKC at these domains even in the absence of the actin cytoskeleton (Figures 3 and 4). While aPKC is also recruited to apical sites outside of membrane domains, this “diffuse” aPKC requires the actin cytoskeleton and rapidly depolarizes in the presence of LatA. Furthermore, when membrane domains are ablated with cyclodextrin, polarity is significantly reduced (Figures 4B and 4E). Thus, we propose that the two pools of membrane-bound aPKC, diffuse and membrane-domain associated, work together to initiate and maintain apical neuroblast polarity (Figure 4G). Given that the membrane density appears to be higher at domain sites (Figure 1), the extended maintenance at domains could arise simply from an initially higher concentration of aPKC. Further work will be necessary to understand if domain-associated aPKC is mechanistically distinct from its diffuse counterpart.

## STAR★METHODS

### RESOURCE AVAILABILITY

#### Lead contact

Further information and requests for resources and reagents should be directed to the Lead Contact, Kenneth Prehoda (prehoda@uoregon.edu).

#### Materials availability

This study did not generate new unique reagents.

#### Data and code availability

The raw data supporting the current study have not been deposited in a public repository because of their large file size but are available from the corresponding author on request.

### EXPERIMENTAL MODEL AND SUBJECT DETAILS

#### Fly strains

For live imaging of membrane dynamics in neuroblasts, a Worniu-Gal4 driver line was used to express fluorescent membrane-bound fusion proteins under UAS control. Three classes of membrane-bound fusion proteins were used. FP-farnesyl expresses the C-terminal region of human K-Ras tagged with GFP which becomes farnesylated and membrane-anchored in cells. FP-PH expresses the pleckstrin homology domain of human PLCδ tagged with GFP or mCherry, which binds to the plasma membrane lipid phosphoinositide PI(4,5)P2. FP-CD8 (a gift from the Chris Doe Lab) expresses a single-pass transmembrane protein tagged with GFP. RFP-His2A expresses RFP-tagged His2A under the control of native promoters. The BAC-encoded GFP-aPKC (Besson et al., 2015) was used to track aPKC localization and dynamics.

### METHOD DETAILS

#### Live imaging

Intact *Drosophila* central nervous systems were dissected from third instar larvae in a bath of Schneider’s Insect Media. These larval brain explants were then mounted dorsal side down on sterile poly-D-lysine coated 35mm glass bottom dishes (ibidi Cat#81156) containing modified minimal hemolymph-like solution (HL3.1). Explants were imaged on a Nikon Eclipse Ti-2 (60x H_2_O objective) equipped with a Yokogawa CSU-W1 SoRa spinning disk head and dual Photometrics Prime BSI sCMOS cameras. GFP tagged proteins were illuminated with 488nm laser light. RFP, mCherry, and mRuby tagged proteins were illuminated with 561nm laser light. For time-lapse imaging 41–61 optical sections with a step size of 0.5 μm were acquired every 20 s. For super resolution imaging, individual neuroblasts were imaged with a step size of 0.3 μm using SoRa optics which achieve super resolution through optical photon reassignment. To examine the role of F-actin in membrane and aPKC dynamics, explants were treated with 50 μM latrunculin A (0.5% DMSO) during imaging. To examine the role of cholesterol in membrane and aPKC dynamics, explants were treated with 15 mM methyl-B-cyclodextrin (solubilized in HL3.1) during imaging. We observed loss of F-actin within minutes of LatA treatment but membrane domains persisted approximately 30 minutes following cyclodextrin treatment, presumably because of cyclodextrin’s larger mass and concomitant slower diffusion into the tissue.

#### Image processing and analysis

Images were analyzed using ImageJ (FIJI package) and Imaris (Bitplane) software. Neuroblasts were identified through their location in the brain and large size, as well as the presence of tissue-specifically expressed transgenes. For rotating movies, maximum intensity projections (MIPs) were assembled from optical slices through the entirety of the neuroblast volume. Photobleaching during time-lapse imaging was corrected for using ImageJ bleach correction tool in histogram matching mode. For aPKC images, a guassian blur of 0.5 pixels was used to improve signal to noise. For imaging of RFP and mCherry tagged transgenes, ImageJ despeckle and/or smooth tools were applied. Kymographs were created from time-lapse movies using a line along the basal-apical axis using ImageJ multi kymograph tool. To quantify the number of membrane domains in the apical and basal hemispheres of the membrane, larval neuroblasts (n = 5) expressing worniu-GAL4 driven GFP-PH were imaged using traditional spinning disk microscopy. Membrane domain were distinguished based on a cutoff of 3X signal intensity above background and an area of at least 0.3 square micrometers. Membrane domains were found to range in size from 0.3–5 square micrometers. To quantify the effect of cyclodextrin on aPKC and Miranda recruitment, movies from larval neuroblasts expressing either GFP-aPKC from its native promoter or worniu-GAL4 driven GFP-Miranda were analyzed. For GFP-aPKC, medial optical slices from post NEB, pre-anaphase cells captured using SoRa spinning disk microscopy were then used to trace the apical cortex and averaged across this region of interest in FIJI. A second region of interest within the cytoplasm was used to determine the apical:cytoplasmic ratio for untreated (n = 13) versus cyclodextrin treated (n = 11) neuroblasts. A similar protocol was used to assess miranda signal intensity along the basal cortex at anaphase onset however medial slices acquired using traditional spinning disk time-lapse imaging were used to trace the basal cortex from which miranda signal intensity was measured and averaged across this region of interest in FIJI. A second region of interest within the cytoplasm was used to determine the basal:cytoplasmic ratio for untreated (n = 7) versus cyclodextrin treated (n = 7) neuroblasts. Particle tracking was performed using the ‘spots’ tracking utility in Imaris. Here, membrane domains were tracked in a neuroblast expressing worniu-GAL4 driven GFP-PH during both the polarization and depolarization phases of the polarity cycle.

### QUANTIFICATION AND STATISTICAL ANALYSIS

Gardner-Altman estimation plots and 95% confidence intervals of cortical intensities were prepared using the DABEST package (Ho et al., 2019). Statistical details can be found in the relevant methods section and figure legend, with each “n” representing a distinct neuroblast.

## Supplementary Material

1

2

3

4

5

6

7

8

9

10

## Figures and Tables

**Table:** KEY RESOURCES TABLE

REAGENT or RESOURCE	SOURCE	IDENTIFIER
Chemicals, peptides, and recombinant proteins		
Latrunculin A	Enzo Life Sciences	Cat#BML-T119-0100, CAS# 76343-93-6
Methyl-β-cyclodextrin	Caymen Chemical	Cat#21633, CAS# 128446-36-6
Experimental models: Organisms/strains		
UAS-aPKC RNAi	Vienna *Drosophila* Resource Center	v105624
FP-Farnesyl	Bloomington *Drosophila* Stock Center	BDSC Cat#80052; RRID:BDSC_80052
FP-PH (GFP)	Bloomington *Drosophila* Stock Center	BDSC Cat#39693; RRID:BDSC_39693
FP-CD8	Bloomington *Drosophila* Stock Center	BDSC Cat#5130; RRID:BDSC_5130
Worniu-Gal4	Chris Q. Doe Lab	N/A
RFP-His2a (H2A)	Bloomington *Drosophila* Stock Center	BDSC Cat#23650; RRID:BDSC_23650
GFP-aPKC	François Schweisguth Lab; (Besson et al., 2015)	PMID: 25843034
FP-PH (mCherry)	Bloomington *Drosophila* Stock Center	BDSC Cat#51658; RRID:BDSC_51658
GFP-Miranda	Bloomington *Drosophila* Stock Center	BDSC Cat#56555; RRID:BDSC_56555
mRuby-Lifeact	Bloomington *Drosophila* Stock Center	BDSC Cat#35545; RRID:BDSC_35545
Baz-GFP	Carnegie Protein Trap Library	PMID: 17194782
Software and algorithms		
ImageJ (FIJI package)	National Institutes of Health	N/A
Imaris	Bitplane	N/A

## Highlights

- *Drosophila* neural stem cell membranes are heterogenous
- Membrane features become polarized during asymmetric division
- Membrane and protein polarity cycles are precisely synchronized
- Actin-dependent forces deform the surrounding tissue during the polarity cycle

## References

[R1] Azuma T, and Kei T (2015). Super-resolution spinning-disk confocal microscopy using optical photon reassignment. Opt. Express 23, 15003–15011. 26072856 10.1364/OE.23.015003

[R2] Barman S, and Nayak DP (2007). Lipid raft disruption by cholesterol depletion enhances influenza A virus budding from MDCK cells. J. Virol 81, 12169–12178. 17855515 10.1128/JVI.00835-07 PMC2169012

[R3] Barros CS, Phelps CB, and Brand AH (2003). Drosophila nonmuscle myosin II promotes the asymmetric segregation of cell fate determinants by cortical exclusion rather than active transport. Dev. Cell 5, 829–840. 14667406 10.1016/s1534-5807(03)00359-9

[R4] Besson C, Bernard F, Corson F, Rouault H, Reynaud E, Keder A, Mazouni K, and Schweisguth F (2015). Planar Cell Polarity Breaks the Symmetry of PAR Protein Distribution prior to Mitosis in Drosophila Sensory Organ Precursor Cells. Curr. Biol 25, 1104–1110. 25843034 10.1016/j.cub.2015.02.073

[R5] Cabernard C, Prehoda KE, and Doe CQ (2010). A spindle-independent cleavage furrow positioning pathway. Nature 467, 91–94. 20811457 10.1038/nature09334 PMC4028831

[R6] Connell M, Cabernard C, Ricketson D, Doe CQ, and Prehoda KE (2011). Asymmetric cortical extension shifts cleavage furrow position in Drosophila neuroblasts. Mol. Biol. Cell 22, 4220–4226. 21937716 10.1091/mbc.E11-02-0173 PMC3216648

[R7] Doyle SE, Pahl MC, Siller KH, Ardiff L, and Siegrist SE (2017). Neuroblast niche position is controlled by Phosphoinositide 3-kinase-dependent DE-Cadherin adhesion. Development 144, 820–829. 28126840 10.1242/dev.136713 PMC5374343

[R8] Gubieda AG, Packer JR, Squires I, Martin J, and Rodriguez J (2020). Going with the flow: insights from *Caenorhabditis elegans* zygote polarization. Philos. Trans. R. Soc. Lond. B Biol. Sci 375, 20190555. 32829680 10.1098/rstb.2019.0555 PMC7482210

[R9] Hannaford MR, Ramat A, Loyer N, and Januschke J (2018). aPKC-mediated displacement and actomyosin-mediated retention polarize Miranda in *Drosophila* neuroblasts. eLife 7, e29939. 29364113 10.7554/eLife.29939 PMC5783611

[R10] Hirani N, Illukkumbura R, Bland T, Mathonnet G, Suhner D, Reymann A-C, and Goehring NW (2019). Anterior-enriched filopodia create the appearance of asymmetric membrane microdomains in polarizing *C. elegans* zygotes. J. Cell Sci 132, jcs230714. 31221727 10.1242/jcs.230714 PMC6679585

[R11] Ho J, Tumkaya T, Aryal S, Choi H, and Claridge-Chang A (2019). Moving beyond P values: data analysis with estimation graphics. Nat. Methods 16, 565–566. 31217592 10.1038/s41592-019-0470-3

[R12] Homem CCF, and Knoblich JA (2012). Drosophila neuroblasts: a model for stem cell biology. Development 139, 4297–4310. 23132240 10.1242/dev.080515

[R13] Illukkumbura R, Bland T, and Goehring NW (2020). Patterning and polarization of cells by intracellular flows. Curr. Opin. Cell Biol 62, 123–134. 31760155 10.1016/j.ceb.2019.10.005 PMC6968950

[R14] Knoblich JA (2010). Asymmetric cell division: recent developments and their implications for tumour biology. Nat. Rev. Mol. Cell Biol 11, 849–860. 21102610 10.1038/nrm3010 PMC3941022

[R15] Koe CT, Tan YS, Lönnfors M, Hur SK, Low CSL, Zhang Y, Kanchanawong P, Bankaitis VA, and Wang H (2018). Vibrator and PI4KIIIα govern neuroblast polarity by anchoring non-muscle myosin II. eLife 7, e33555. 29482721 10.7554/eLife.33555 PMC5828666

[R16] Lang CF, and Munro E (2017). The PAR proteins: from molecular circuits to dynamic self-stabilizing cell polarity. Development 144, 3405–3416. 28974638 10.1242/dev.139063 PMC5665476

[R17] Lee T, and Luo L (1999). Mosaic analysis with a repressible cell marker for studies of gene function in neuronal morphogenesis. Neuron 22, 451–461. 10197526 10.1016/s0896-6273(00)80701-1

[R18] Loyer N, and Januschke J (2018). The last-born daughter cell contributes to division orientation of Drosophila larval neuroblasts. Nat. Commun 9, 3745. 30218051 10.1038/s41467-018-06276-0 PMC6138640

[R19] Munro E, Nance J, and Priess JR (2004). Cortical flows powered by asymmetrical contraction transport PAR proteins to establish and maintain anterior-posterior polarity in the early C. elegans embryo. Dev. Cell 7, 413–424. 15363415 10.1016/j.devcel.2004.08.001

[R20] Oon CH, and Prehoda KE (2019). Asymmetric recruitment and actin-dependent cortical flows drive the neuroblast polarity cycle. eLife 8, e45815. 31066675 10.7554/eLife.45815 PMC6524966

[R21] Pang DJ, Hayday AC, and Bijlmakers M-J (2007). CD8 Raft localization is induced by its assembly into CD8α β heterodimers, Not CD8α α homodimers. J. Biol. Chem 282, 13884–13894. 17341584 10.1074/jbc.M701027200

[R22] Petronczki M, and Knoblich JA (2001). DmPAR-6 directs epithelial polarity and asymmetric cell division of neuroblasts in Drosophila. Nat. Cell Biol 3, 43–49. 11146625 10.1038/35050550

[R23] Pohl C, Tiongson M, Moore JL, Santella A, and Bao Z (2012). Actomyosin-based self-organization of cell internalization during C. elegans gastrulation. BMC Biol 10, 94. 23198792 10.1186/1741-7007-10-94 PMC3583717

[R24] Riga A, Castiglioni VG, and Boxem M (2020). New insights into apical-basal polarization in epithelia. Curr. Opin. Cell Biol 62, 1–8. 31505411 10.1016/j.ceb.2019.07.017

[R25] Roh-Johnson M, Shemer G, Higgins CD, McClellan JH, Werts AD, Tulu US, Gao L, Betzig E, Kiehart DP, and Goldstein B (2012). Triggering a cell shape change by exploiting preexisting actomyosin contractions. Science 335, 1232–1235. 22323741 10.1126/science.1217869 PMC3298882

[R26] Rolls MM, Albertson R, Shih H-P, Lee C-Y, and Doe CQ (2003). Drosophila aPKC regulates cell polarity and cell proliferation in neuroblasts and epithelia. J. Cell Biol 163, 1089–1098. 14657233 10.1083/jcb.200306079 PMC2173607

[R27] Roth M, Roubinet C, Iffländer N, Ferrand A, and Cabernard C (2015). Asymmetrically dividing Drosophila neuroblasts utilize two spatially and temporally independent cytokinesis pathways. Nat. Commun 6, 6551. 25791062 10.1038/ncomms7551 PMC4544045

[R28] Roubinet C, Tsankova A, Pham TT, Monnard A, Caussinus E, Affolter M, and Cabernard C (2017). Spatio-temporally separated cortical flows and spindle geometry establish physical asymmetry in fly neural stem cells. Nat. Commun 8, 1383. 29123099 10.1038/s41467-017-01391-w PMC5680339

[R29] Scholze MJ, Barbieux KS, De Simone A, Boumasmoud M, Süess CCN, Wang R, and Gönczy P (2018). PI(4,5)P2 forms dynamic cortical structures and directs actin distribution as well as polarity in Caenorhabditis elegans embryos. Development 145, dev164988. 29724757 10.1242/dev.164988

[R30] Stone MB, Shelby SA, and Veatch SL (2017). Super-Resolution Microscopy: Shedding Light on the Cellular Plasma Membrane. Chem. Rev 117, 7457–7477. 28211677 10.1021/acs.chemrev.6b00716 PMC5471115

[R31] Sunchu B, and Cabernard C (2020). Principles and mechanisms of asymmetric cell division. Development 147, dev167650. 32601056 10.1242/dev.167650 PMC7338270

[R32] Tsankova A, Pham TT, Garcia DS, Otte F, and Cabernard C (2017). Cell Polarity Regulates Biased Myosin Activity and Dynamics during Asymmetric Cell Division via Drosophila Rho Kinase and Protein Kinase N. Dev. Cell 42, 143–155.e5. 28712722 10.1016/j.devcel.2017.06.012

[R33] van IJzendoorn SCD, Agnetti J, and Gassama-Diagne A (2020). Mechanisms behind the polarized distribution of lipids in epithelial cells. Biochim. Biophys. Acta Biomembr 1862, 183145. 31809710 10.1016/j.bbamem.2019.183145

[R34] Venkei ZG, and Yamashita YM (2018). Emerging mechanisms of asymmetric stem cell division. J. Cell Biol 217, 3785–3795. 30232100 10.1083/jcb.201807037 PMC6219723

[R35] Verstreken P, Ohyama T, Haueter C, Habets RLP, Lin YQ, Swan LE, Ly CV, Venken KJT, De Camilli P, and Bellen HJ (2009). Tweek, an evolutionarily conserved protein, is required for synaptic vesicle recycling. Neuron 63, 203–215. 19640479 10.1016/j.neuron.2009.06.017 PMC2759194

[R36] Wang S-C, Low TYF, Nishimura Y, Gole L, Yu W, and Motegi F (2017). Cortical forces and CDC-42 control clustering of PAR proteins for Caenorhabditis elegans embryonic polarization. Nat. Cell Biol 19, 988–995. 28737772 10.1038/ncb3577

[R37] Wodarz A, Ramrath A, Grimm A, and Knust E (2000). Drosophila atypical protein kinase C associates with Bazooka and controls polarity of epithelia and neuroblasts. J. Cell Biol 150, 1361–1374. 10995441 10.1083/jcb.150.6.1361 PMC2150710

[R38] Zhou Y, Wong C-O, Cho KJ, van der Hoeven D, Liang H, Thakur DP, Luo J, Babic M, Zinsmaier KE, Zhu MX, (2015). SIGNAL TRANSDUCTION. Membrane potential modulates plasma membrane phospholipid dynamics and K-Ras signaling. Science 349, 873–876. 26293964 10.1126/science.aaa5619 PMC4687752

